# War Bread

**Published:** 1918-04-27

**Authors:** 


### War Bread.

The Food (War) Committee of the Royal Society has issued its report on this subject. The problems considered were: 1. (*a*) What gain, if any, in food value results from raising the milling standard of wheat from 80 to 90 per cent.? (*b*) Does the dilution of bread with other cereals than wheat diminish its food value? 2. What is the effect of the consumption of these breads on the health of the consumers? 3. Is such bread likely to be acceptable? The trials were carried out on three groups of persons in different parts of the country, and the results were remarkably uniform in the different groups, and even in the different individuals in each group. Of the 80 per cent. bread 96.1 per cent. was extracted in digestion; of the 90 per cent. bread only 94.5 per cent. was extracted, giving a net gain to the 90 per cent. bread of 8.17 per cent., so that a supply of flour which, milled to 80 per cent., would last the country for a year, would, if milled to 90 per cent., last thirteen months.

A second set of experiments was made with bread consisting of four-fifths wheat milled to 80 per cent., and one-fifth maize flour. At first there was a little disturbance of digestion with this bread, sometimes diarrhœa, more often constipation; but this soon passed off. The bread was found equally digestible with bread made from wheat alone, and there was no difference in the proportion of energy in the bread that was extracted for the use of the body.

The next trial was with bread made as to four-fifths of 90 per cent. wheat, the remaining fifth being half barley and half rice, or half barley, a quarter rice, and a quarter maize. This bread caused no digestive trouble and was well liked, so much so that the persons experimented on begged that it might be continued. It was not less nutritious than bread made wholly of wheat. Bread made from mixed flour gives a rather larger residue, which may irritate the bowel that suffers from colitis; but to healthy people is rather an advantage than otherwise.

So far, it appears that the changes already made in our bread in consequence of the war have been rather advantageous to health than otherwise, and that further changes in the direction of dilution of wheat flour with the flour of other cereals may be contemplated with equanimity.

